# The Clinical Significance of Attached Gingiva in the Natural Dentition

**DOI:** 10.3390/dj14030156

**Published:** 2026-03-09

**Authors:** João Carnio, João Kreling Carnio, Paulo M. Camargo

**Affiliations:** 1 Private Practice, Adhemar Pereira de Barros 131, Londrina 86050-190, PR, Brazil; 2 School of Dentistry, University of Cesumar (UniCesumar), Londrina 86027-810, PR, Brazil; jkcarnio@gmail.com; 3 Section of Periodontics, UCLA School of Dentistry, Los Angeles UCLA, Los Angeles, CA 90024, USA; pcamargo@dentistry.ucla.edu

**Keywords:** attached gingiva, keratinized tissue, mucogingival therapy, supracrestal tissue attachment, gingival recession

## Abstract

**Background:** The attached gingiva (AG) is the portion of the gingiva firmly bound to the underlying alveolar bone and root cementum, rendering it immobile during functioning. Its dense connective tissue attachment contributes to resistance against mechanical challenges, stabilization of the gingival margin, and dissipation of forces transmitted from the alveolar mucosa. Histologically, AG is characterized by a keratinized epithelium supported by dense collagen fiber bundles, which provide structural integrity to the dento–gingival unit. Clinically, the buccal and lingual width of AG is estimated by subtracting sulcus depth from the total width of keratinized tissue. Although periodontal health may be maintained with minimal AG under optimal plaque control, substantial evidence supports its role in preserving gingival architecture and resisting mechanical trauma. **Practical Application:** From a clinical perspective, an adequate width of attached gingiva has traditionally been considered necessary to protect the periodontium; however, clinical situations may exist in which its dimension is reduced or absent. Available evidence suggests that a minimal width of approximately 1 mm of attached gingiva may be sufficient to maintain periodontal health under conditions of effective plaque control and absence of inflammation. Nevertheless, when only this minimal dimension is present, the attachment is predominantly derived from the junctional epithelium, which may offer limited mechanical protection to the dento–gingival unit. Within the limits of current evidence, a keratinized tissue width of approximately 3 mm appears to represent a functional threshold associated with increased connective tissue fiber density and enhanced resistance to mechanical trauma. **Methods:** A narrative review of classical and contemporary literature was conducted to evaluate the morphology, histology, function, and clinical relevance of the attached gingiva. **Results:** Evidence indicates that when AG width is minimal, reliance on junctional epithelial attachment alone provides limited resistance to mechanical challenges. In contrast, a greater width of AG incorporating connective tissue fiber attachment is associated with improved gingival margin stability, enhanced mechanical protection, and periodontal tissue resilience. Based on this synthesis, a tissue-based clinical categorization of AG is proposed. **Conclusions:** This review integrates current biological and clinical concepts regarding the functional dimensions of attached gingiva. The proposed categorization offers a practical framework to support clinical decision-making and to identify conditions in which surgical augmentation may be indicated for the management of mucogingival deficiencies.

## 1. Introduction

The attached gingiva (AG) is the portion of the gingiva that is firm, dense, stippled, and tightly bound to the underlying periosteum, tooth, and alveolar bone. It is composed of keratinized epithelium (KT) supported by dense connective tissue, largely filled with tightly packed collagen fiber bundles [1].

Apically, the gingiva is continuous with the non-keratinized alveolar mucosa, which is mobile and loosely bound to the underlying tooth and bone. The demarcation line between the gingiva and the alveolar mucosa is termed the mucogingival junction [1].

Histologically, on its coronal aspect, the junctional epithelium (JE), which represents the initial contact between the gingiva and the tooth, forms the epithelial attachment [2]. Apical to the JE, connective tissue fibers embedded in the root cementum constitute the connective tissue attachment to the tooth [3].

In permanent dentition, the width of the AG varies considerably, ranging from approximately 1 to 9 mm [4,5,6].

Clinically, the buccal and lingual width of AG is estimated by subtracting the sulcus or pocket depth from the distance between the gingival margin and the mucogingival junction. This method inherently includes the junctional epithelium within the clinical measurement of AG, despite the distinct structural and functional characteristics of epithelial and connective tissue attachments [2]. Consequently, the proportion of JE included in this measurement may vary depending on probing depth and inflammatory status. Recognition of these distinctions is essential when interpreting the functional significance of AG in periodontal stability [7].

Carnio and co-workers proposed that the AG enhances resistance of the periodontium to external injury, contributes to stabilization of the gingival margin, and assists in dissipating physiological forces transmitted by the muscular fibers of the alveolar mucosa to the gingival tissues [8].

From a clinical perspective, several studies have suggested that a minimal width of AG is required to maintain periodontal health [9,10,11,12,13,14,15]. Lang and Löe (1972), and more recently Scheyer et al. (2015), recommended a minimum of 2 mm of keratinized tissue, including at least 1 mm of attached gingiva, particularly in patients with suboptimal plaque control [16,17]. However, subsequent investigations have challenged this requirement, demonstrating that periodontal health may be preserved even in sites with minimal or absent AG, provided that inflammation and traumatic factors are adequately controlled [18,19].

The average supracrestal tissue attachment (SCTA), previously referred to as the biologic width, consists of approximately 1 mm of epithelial attachment and 1 mm of connective tissue attachment. These dimensions have been histologically confirmed under healthy conditions and serve as important reference parameters for periodontal and restorative treatment planning [20,21,22].

When the collective evidence is considered, it becomes apparent that in the presence of 2 mm of KT, the adherence corresponding to the AG consists predominantly of junctional epithelial attachment rather than dense connective tissue attachment [16,20,21,22,23,24].

Schroeder and Listgarten described the JE as a specialized anatomical structure that facilitates the transmigration of neutrophils and the transient influx of mononuclear leukocytes, playing a critical role in host defense against bacterial challenge [25]. However, this attachment, mediated primarily through hemidesmosomes, is biologically fragile and poorly suited to resist mechanical forces exerted by the alveolar mucosa on the gingival margin [24,26]. Although the epithelial attachment is essential for protection against biofilm accumulation, its defensive role does not appear to depend on the width of keratinized tissue present [16,18,27,28,29].

Waerhaug demonstrated that insertion of dental floss into the base of the sulcus and repeated movement against the tooth surface resulted in detachment of junctional epithelial cells [30]. Similarly, periodontal probing has been shown to disrupt the JE even in clinically non-inflamed sites [31]. The limited number of desmosomes between junctional epithelial cells, together with variability in intercellular space dimensions, helps explain why the epithelial attachment represents a relatively weak barrier against mechanical trauma [2,21,30].

**Figure 1:**
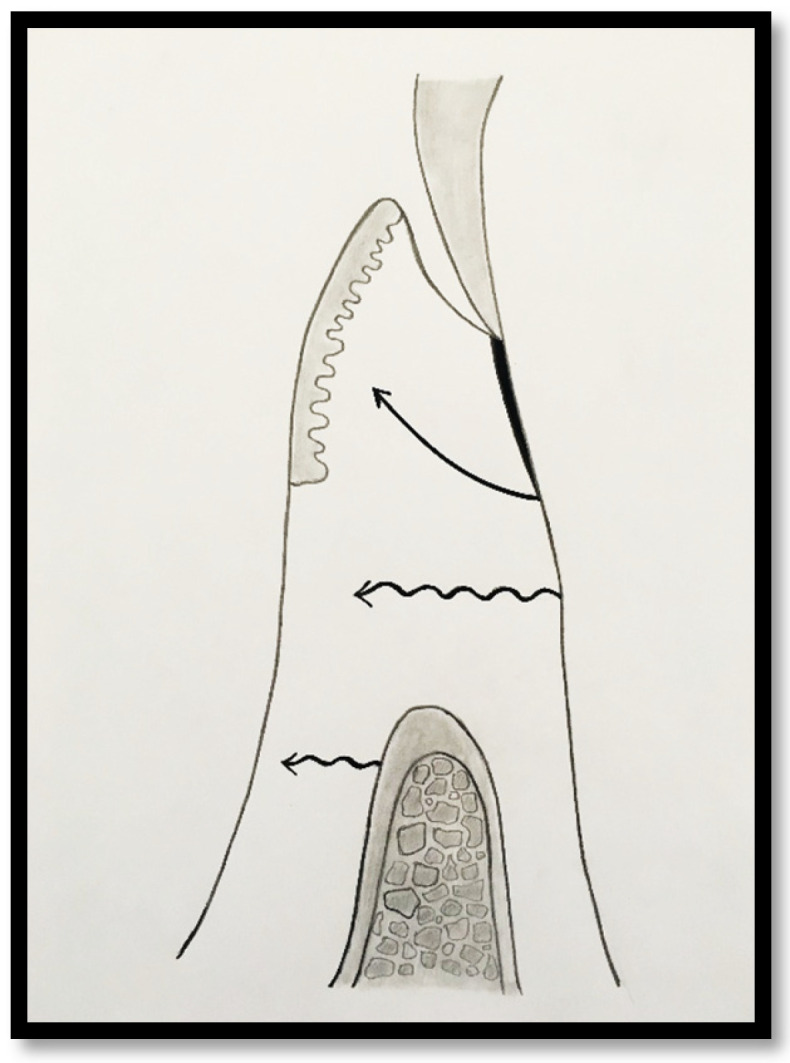
Probing depth of 1 mm is associated with approximately 1 mm of epithelial attachment.

**Figure 2:**
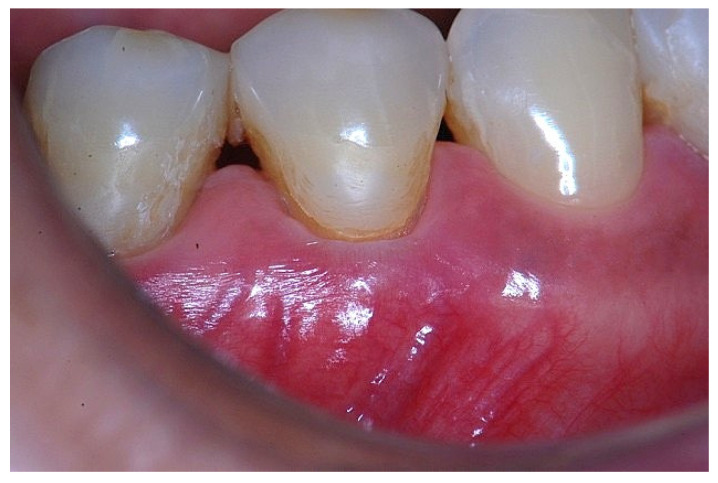
Clinical view of tooth #29 showing an insufficient width of attached gingiva on the facial aspect.

**Figure 3:**
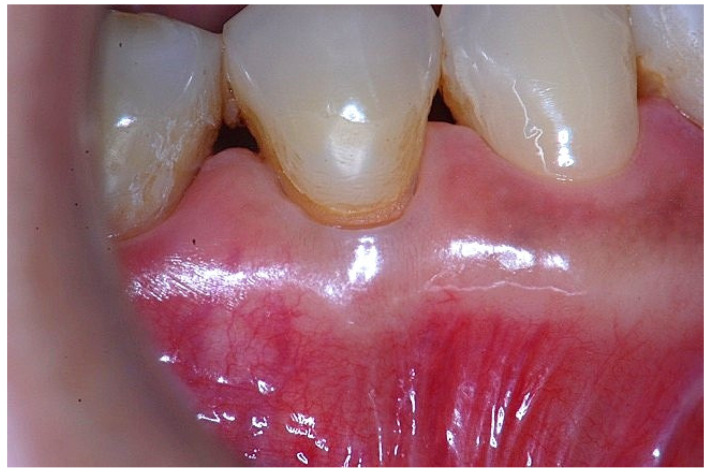
Apical displacement of the gingival margin observed during deep lip retraction.

**Figure 4:**
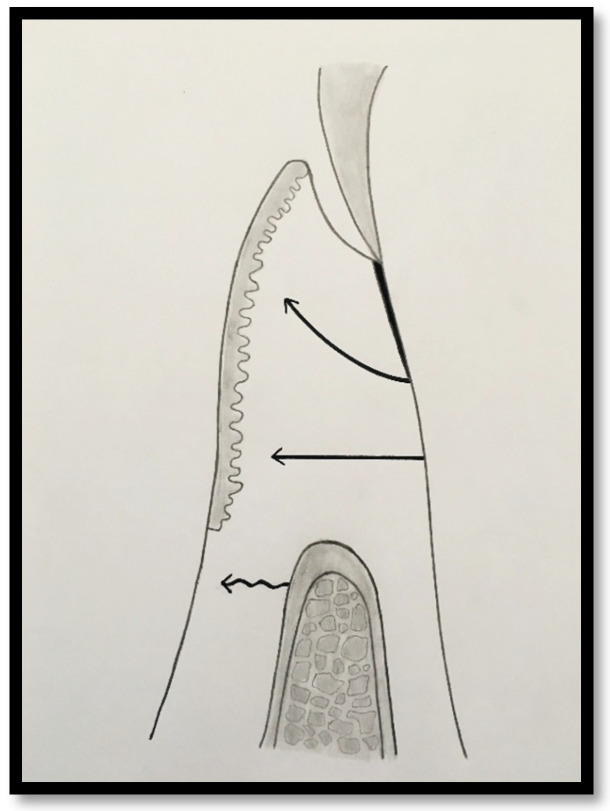
Probing depth of 1 mm associated with 1 mm of epithelial attachment and 1 mm of connective tissue fiber insertion into the root cementum.

**Figure 5:**
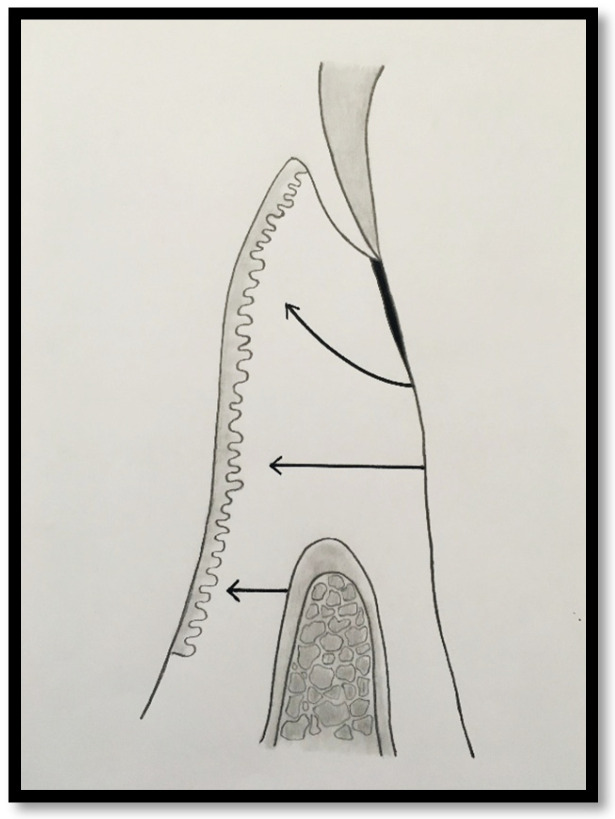
Probing depth of 1 mm associated with 1 mm of epithelial attachment, 1 mm of connective tissue fiber insertion into the root cementum, and connective tissue fibers inserting into the alveolar bone.

**Figure 6:**
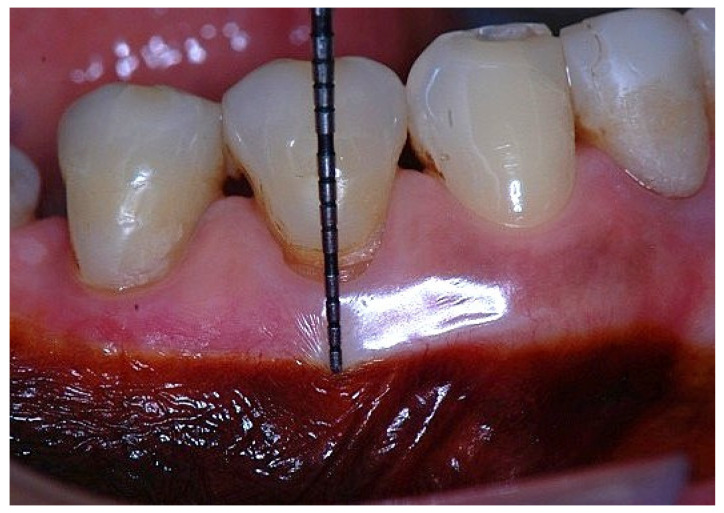
Surgical establishment of a new zone of approximately 4 mm of attached gingiva.

In a clinical scenario characterized by 2 mm of keratinized tissue and a sulcus depth of 1 mm, a stable and mechanically resistant connective tissue attachment is not present [1]. This is attributable to the fact that most connective tissue fibers inserting into the root surface are associated with non-keratinized, mobile mucosal tissues (Figure 1), which are elastic in nature and therefore incapable of stabilizing the gingival margin (Figure 2 and Figure 3). In contrast, when these fibers are embedded within keratinized tissue, they are firmly anchored to both the tooth and alveolar bone and are comparatively immobile, thereby contributing to gingival margin stability (Figure 4, Figure 5 and Figure 6).

Accordingly, while 2 mm of keratinized tissue may be sufficient for maintenance of periodontal health in patients with optimal oral hygiene, this dimension may be inadequate to stabilize the gingival margin against external mechanical challenges [16,17,32].

To achieve predictable resistance to mechanical trauma, a keratinized tissue width of approximately 3 mm appears necessary, assuming a sulcus depth of 1 mm, thereby allowing for 1 mm of epithelial attachment and 1 mm of dense connective tissue attachment to the root surface [21,33].

**Figure 7:**
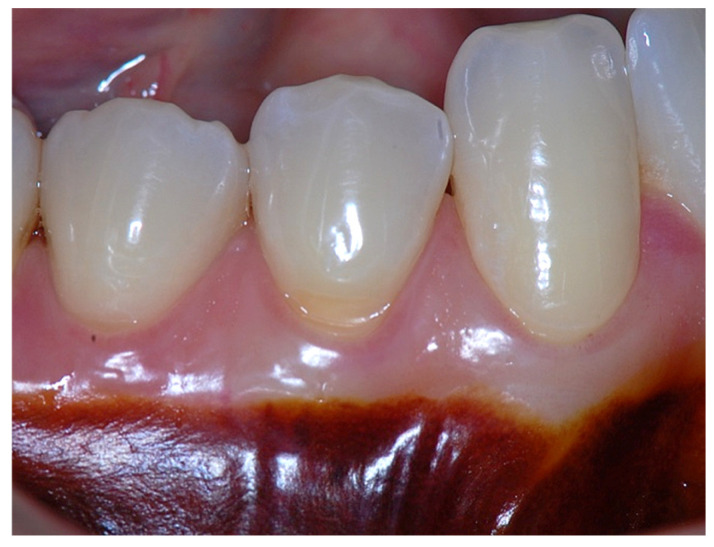
Clinical presentation of a 44-year-old patient with progressive gingival recession affecting tooth #28.

**Figure 8:**
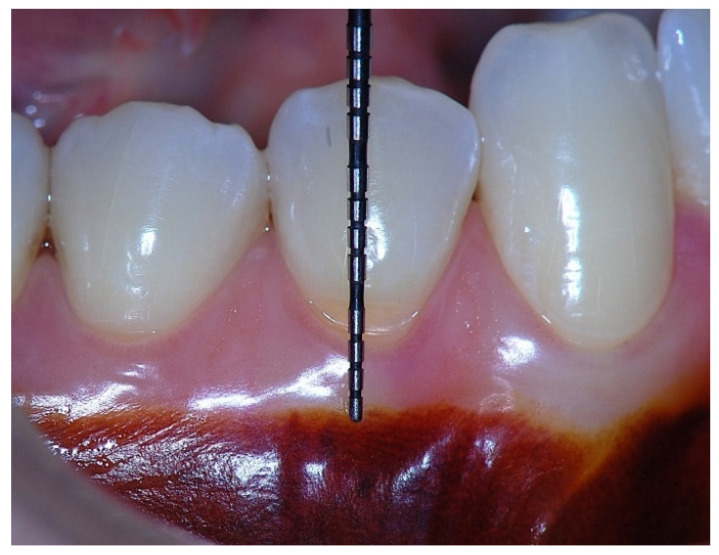
Clinical view showing a 2 mm band of keratinized tissue on the facial aspect.

**Figure 9:**
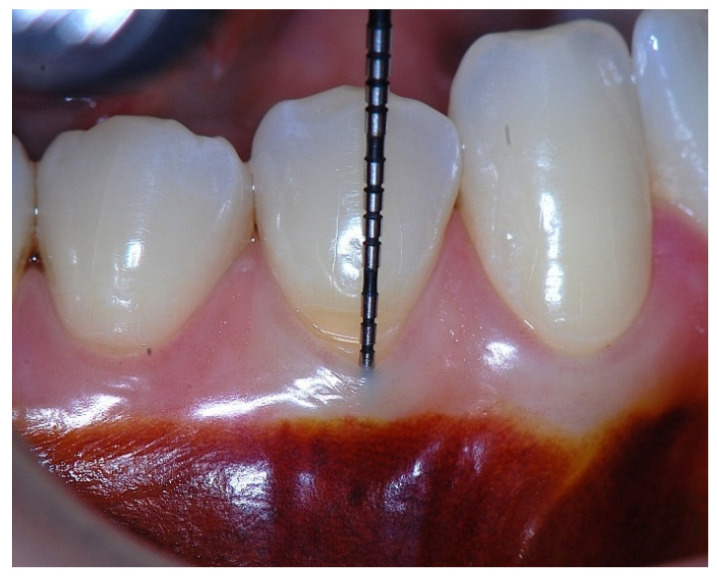
Probing depth of approximately 1 mm illustrating that a narrow band of attached gingiva corresponds predominantly to epithelial attachment.

**Figure 10:**
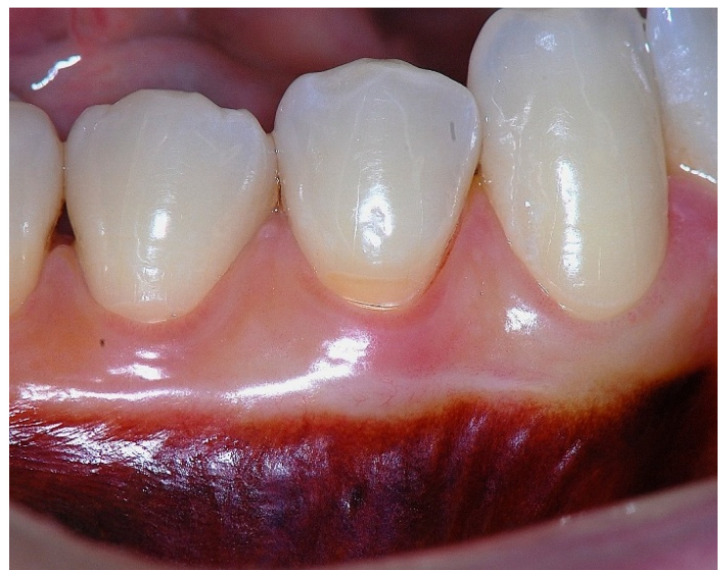
A surgical procedure performed to increase the width of keratinized and attached gingiva.

**Figure 11:**
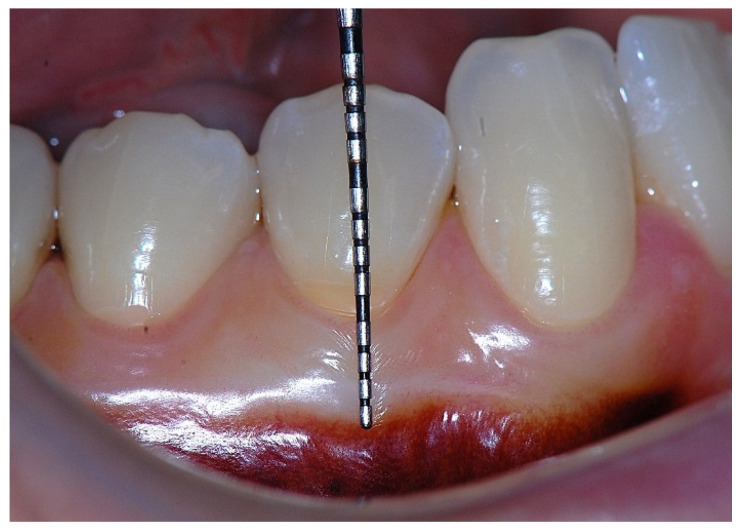
Three-year postoperative clinical outcome showing a stable 4 mm zone of keratinized tissue, corresponding to approximately 3 mm of attached gingiva.

Clinically, an ideal scenario involves a keratinized tissue width of ≥3 mm, as this configuration supports connective tissue fiber insertion into both the root surface and the periosteum of the alveolar bone (Figure 7, Figure 8, Figure 9, Figure 10 and Figure 11). Notably, a KT width ≥3 mm represents the only condition that fully aligns with classical definitions of attached gingiva in the periodontal literature and with American Academy of Periodontology criteria, which describe AG as being firmly attached to the underlying alveolar bone and root cementum by dense connective tissue fibers [1,3,34,35].

## 2. Materials and Methods

A narrative literature review was conducted to summarize and critically analyze existing knowledge regarding the morphology, biological concepts, and clinical relevance of the amount of attached gingiva (AG) in natural dentition. A comprehensive bibliographic search was performed in the PubMed/MEDLINE, Scopus, and SciELO databases, including articles published from 1961 to the present.

The search strategy combined the following terms: attached gingiva, keratinized tissue, gingival morphology, gingival width, gingival thickness, periodontal stability, gingival recession, and periodontal health. Additional relevant publications were identified through manual screening of the reference lists of selected articles.

Inclusion criteria comprised original research articles, clinical studies, and narrative or systematic reviews addressing the morphology, histology, function, or clinical relevance of attached gingiva in natural dentition, with priority given to classical and landmark investigations that established fundamental biological and clinical concepts. Exclusion criteria included in vitro studies, case reports involving fewer than three subjects, studies focusing exclusively on implant-related keratinized tissue without relevance to natural dentition, and publications lacking sufficient methodological detail or clinically relevant data.

The selected literature was screened and analyzed based on its relevance to (1) the historical evolution of AG concepts; (2) clinical and histological assessment methods; (3) functional and protective roles of attached gingiva; and (4) implications for periodontal stability, mechanical trauma, and treatment outcomes. Data were analyzed descriptively and thematically.

It is important to note that width-based clinical measurements of attached gingiva are anatomically applicable primarily to facial and lingual surfaces. In interproximal sites, a corono-apical mucogingival junction is absent, and the keratinized epithelium forms a continuous circumferential collar around the tooth. As a result, the linear concept of “attached gingiva width” is not anatomically defined in these regions, where connective tissue architecture is predominantly characterized by transseptal and interdental fibers rather than alveolo-gingival fibers. Accordingly, the tissue-based framework and dimensional thresholds proposed in this review should be interpreted as applicable to buccal and lingual aspects. In interproximal areas, clinical assessment should instead emphasize parameters such as papilla height, col morphology, and overall soft tissue volume rather than mucogingival junction–based width measurements [3].

To ensure clinical relevance, contemporary publications were incorporated to complement foundational histological studies, allowing the discussion to reflect current periodontal and restorative concepts while preserving biological context.

## 3. Clinical Presentation

Based on current evidence and the biological characteristics of the supracrestal buccal and lingual tissue attachment (SCTA) that constitute the dento–gingival unit, the attached gingiva (AG) may be clinically categorized into the following anatomical patterns (A-B-C) [20].

A. Attached Gingiva Predominantly Associated with Epithelial Attachment

In this condition, the attached gingiva consists primarily of junctional epithelium (JE), with only minimal extension of connective tissue fibers into the coronal portion of the keratinized papilla. Most connective tissue fibers, including supracrestal fibers, course apically and insert into the alveolar mucosa rather than into the keratinized tissue. Consequently, the soft tissue attachment in this configuration exhibits limited mechanical resistance and a reduced capacity to stabilize the gingival margin.

B. Attached Gingiva Comprising Epithelial Attachment and Connective Tissue Fibers Inserted into Root Cementum

In this anatomical configuration, connective tissue fibers extend into the keratinized tissue, allowing the attached gingiva to comprise both junctional epithelial attachment and connective tissue fiber insertion into the root cementum. However, supracrestal connective tissue fibers associated with the alveolar bone continue to insert into the alveolar mucosa. This arrangement provides greater mechanical stability compared with the previous category, although resistance to functional and traumatic forces may remain limited.

C. Attached Gingiva Comprising Epithelial Attachment and Connective Tissue Fibers Inserted into Both Root Cementum and Alveolar Bone

This category represents the most favorable anatomical and functional condition. In this scenario, both connective tissue fibers inserting into the root cementum and supracrestal fibers associated with the alveolar bone extend into the keratinized tissue. This structural organization provides maximal resistance to mechanical forces and optimal stabilization of the gingival margin, thereby offering the highest level of protection against trauma and gingival recession.

## 4. Discussion

The keratinized tissue (KT) and attached gingiva (AG) thresholds discussed herein are intended as reference values for stable periodontal conditions and should not be interpreted as compensatory or protective parameters in the presence of altered attachment quality (e.g., long junctional epithelium), anatomical deficiencies such as fenestrations or dehiscences, extreme biological disruption (e.g., intentional replantation), or excessive biomechanical loading. In such scenarios, the presence of KT may facilitate plaque control and contribute to marginal tissue stability; however, it cannot biologically substitute for compromised attachment or structural support.

The anatomy-related clinical situations of attached gingiva presented in this review are based on the buccal and lingual dimensional characteristics of the supracrestal tissue attachment (SCTA), which comprises both the junctional epithelium and the connective tissue attachment. These anatomical parameters have long served as fundamental references for periodontal and restorative decision-making [16,20,21,36,37,38].

Beyond SCTA dimensions, the orientation and insertion pattern of supracrestal connective tissue fibers relative to the keratinized tissue play a critical role in gingival margin stability, provided that anatomical landmarks remain within physiological limits, namely approximately 1 mm of epithelial attachment and 1 mm of probing depth (Figure 8, Figure 9, Figure 10 and Figure 11).

It is well established that supracrestal connective tissue fibers of the dento–gingival unit are required to extend into dense connective tissue covered by keratinized epithelium on the outer gingival surface. This extension is essential for achieving the protective and resistant functions necessary for maintaining gingival margin stability [39].

Despite this biological understanding, considerable disagreement persists regarding the ideal apico-coronal dimension of the attached gingiva [28,29]. A major source of confusion originates from the long-standing interpretation of the classic study by Lang and Löe, which has frequently been cited as evidence that a minimum of 2 mm of keratinized tissue is required to maintain periodontal health, particularly in individuals with inadequate plaque control [16]. In their study, 80% of sites with ≥2 mm of KT remained clinically healthy, whereas sites with <2 mm exhibited signs of inflammation, leading to the conclusion that 2 mm of KT was sufficient under those conditions.

In contrast, Maynard and Wilson proposed that when restorative margins are placed within the gingival sulcus, a minimum of 5 mm of keratinized tissue—of which at least 3 mm should be attached—is necessary [13]. Their rationale was that AG functions not only as a barrier against plaque-induced inflammation but also as a protective structure against mechanical trauma associated with restorative procedures and oral hygiene practices [14].

Histologic evidence indicates that supracrestal connective tissue fibers may insert into keratinized tissue even when KT is less than 3 mm; however, under such conditions, fiber density and length are generally reduced. From a clinical perspective, a KT width of approximately 3 mm should therefore be interpreted not as an absolute anatomical prerequisite for fiber insertion, but as a functional threshold more likely to ensure sufficient connective tissue density to provide effective mechanical protection of the gingival margin.

These divergent viewpoints reflect the coexistence of two distinct biological concepts: (1) the role of keratinized tissue in modulating susceptibility to plaque-induced inflammation, and (2) its contribution to the mechanical resistance of the gingival tissues against trauma-induced inflammation, such as that caused by aggressive toothbrushing or dental interventions. While approximately 2 mm of KT may be sufficient to maintain periodontal health under optimal conditions, this dimension may be inadequate when mechanical trauma is considered [13,15,17,27,40].

This interpretation is closely related to the biological nature of the soft-tissue attachment. A KT width of approximately 2 mm is often associated with a predominance of epithelial attachment, which, although biologically functional, may not provide sufficient protection to the gingival margin under traumatic conditions. Conversely, a KT width of approximately 3 mm allows connective tissue fibers attached to the root cementum to contribute more effectively to the protective complex, enhancing gingival margin stability, particularly when insertion into the alveolar bone is also present [1,22]. Although this configuration represents a biologically favorable scenario, it should not be interpreted as a universal clinical requirement.

Schroeder described the gingiva as a “collar of masticatory mucosa” attaching to the tooth, alveolar crest, interdental septa, and coronal alveolar process [26]. This protective function, however, may be less effective when only 2 mm of KT is present, particularly on the buccal aspect.

Despite ongoing debate regarding the minimal width of AG required for periodontal stability, there is general clinical consensus that a wider band of KT/AG facilitates oral hygiene, supports maintenance of clinical attachment, and reduces the risk of gingival recession. Evidence further suggests that in patients with inadequate plaque control, increased KT/AG width is associated with reduced inflammation and less attachment loss [12,41]. Consequently, surgical augmentation of attached gingiva remains a predictable and widely accepted therapeutic approach in appropriately selected cases [42,43,44,45].

Finally, an insufficient width of attached gingiva, in combination with the histological characteristics of epithelial and connective tissue attachments, may represent a contributing risk factor for the initiation and progression of gingival recession, particularly in the presence of mechanical trauma. The present article adopts a narrative, concept-oriented approach without a systematic protocol, formal quality assessment, or meta-analysis; therefore, selection bias cannot be excluded. The clinical cases presented are illustrative rather than generalizable, while still allowing integration of classical and contemporary evidence and discussion of persistent biological and clinical controversies.

## 5. Conclusions

This review provides a structured and biologically grounded interpretation of the possible relationships between the attached gingiva (AG) and the underlying periodontal tissues, based on classical and contemporary studies that have shaped current concepts in dentistry and are consistently supported by clinical observation. One of the main sources of controversy regarding the amount and clinical necessity of AG appears to reside in the nature of the attachment being evaluated. In this context, a clinically measured AG width of approximately 1 mm may largely represent junctional epithelial attachment rather than dense connective tissue anchorage.

Under such conditions, careful and atraumatic oral hygiene may allow maintenance of a stable gingival margin for a period of time. However, available evidence consistently supports that the presence of attached gingiva is biologically and clinically preferable to its absence. From a practical standpoint, a keratinized tissue width of approximately 3 mm should be interpreted as a functional threshold associated with greater connective tissue fiber density and enhanced mechanical protection of the gingival margin, rather than as a rigid anatomical requirement.

The concepts summarized in this review provide clinicians with a practical and biologically oriented framework to assess the quality and dimensions of the attached gingiva and to determine, when indicated, whether surgical augmentation procedures may be beneficial for achieving long-term periodontal health and tissue stability.

## 6. Clinical Relevance

Understanding the anatomical variations and biological characteristics of the attached gingiva is essential for accurate clinical evaluation and treatment planning. Recognizing the limits of functional stability in areas with reduced AG allows clinicians to make informed decisions regarding the need for surgical augmentation, thereby contributing to the long-term preservation of periodontal health, tissue stability, and esthetics.

## 7. Future Perspectives

Future investigations employing standardized clinical methodologies and well-defined histological criteria are needed to further elucidate the minimal width and biological requirements of attached gingiva necessary to sustain periodontal health. Such studies may provide deeper insight into the interactions among the junctional epithelium, connective tissue attachment, and keratinized mucosa. A more comprehensive understanding of these relationships will contribute to refining clinical parameters for diagnosing mucogingival deficiencies and will support more evidence-based decision-making regarding the indications for surgical augmentation procedures.

## Data Availability

No new datasets were generated or analyzed during the current study. Therefore, data sharing is not applicable to this article.
